# Complete genome sequence of *Alloalcanivorax xenomutans* HF10, an alkane-degrading bacterium under hypersaline conditions

**DOI:** 10.1128/mra.00050-24

**Published:** 2024-07-31

**Authors:** Jie Zheng, Bo Yu

**Affiliations:** 1 Department of Microbial Physiological & Metabolic Engineering, State Key Laboratory of Mycology, Institute of Microbiology, Chinese Academy of Sciences, Beijing, China; 2 University of Chinese Academy of Sciences, Beijing, China; California State University San Marcos, San Marcos, California, USA

**Keywords:** alkane-degrading bacteria, *Alloalcanivorax xenomutans*, hypersaline

## Abstract

We report the complete genome sequence of *Alloalcanivorax xenomutans* HF10, an alkane-degrading strain isolated from the sediments of ocean in Xiamen, China, with a high salt tolerance potential of more than 10%. Its genome is composed of a 4.76-Mb chromosome.

## ANNOUNCEMENT

*Alcanivorax* is a genus from the Alcanivoracaceae family of the class γ-proteobacteria, the members of which can often degrade a wide spectrum of linear and branched alkanes (1). Members of this genus are Gram-negative, halophilic, and aerobic and have been found to use aliphatic hydrocarbons as the sole source of carbon and energy (2).

*Alloalcanivorax xenomutans* HF10 was isolated from harbor sediments in Xiamen (118°04' East, 24°27' North), China, in June 2022 by the dilution plating method. The sediment was collected by filling 50-mL sampling bottles, and the sediment was diluted with MSM medium containing 3.5% NaCl supplemented with 10 g/L *n*-alkanes as the carbon source and cultured at 30°C, following three times of subculturing every 72 hours. Single colonies were isolated on MSM agar plates. The pure strain was obtained by repeated streaking on the same medium agar plates. The strain was deposited in the China General Microbiological Culture Collection Center (CGMCC No. 25702). Genomic DNA was prepared from an overnight culture from a single colony by using the TIANamp Bacteria DNA kit (TIANGEN, China). Genomic DNA was randomly fragmented by Covaris. The DNBSEQ library was prepared by using the Agencourt AMPure XP-Medium kit to an average size of 200–400 bp. Fragments were end-repaired, 3’-adenylated, and then ligated with primers. Fragments were amplified by polymerase chain reaction (PCR) and heat-denatured and circularized using the splint oligonucleotide sequence. The library was then amplified with phi29 to create DNA nanoballs (DNBs), which were subsequently placed on a patterned nanoarray. Sequencing involved generating 150-base reads from each end using a combinatorial probe-anchor synthesis (cPAS) on a DNBSeq-G400 platform. The Nanopore library was prepared by the Nanopore SQK-RAD114 Kit (Oxford Nanopore Technologies, Britain). Whole-genome sequencing was individually performed on the MGISEQ-2000RS by combined probe anchoring polymerization (cPAS) and Nanopore PromethION platform default chip R9 by Shenzhen BGI Co. LTD, China. The sequencing generated 8,764,210 raw reads, which comprised 4,341,220 paired-end reads (2 × 150 bp) with a depth of 273 × by DNBSEQ. Nanopore platforms generated 109,918 raw reads with an average length of 18,356 bp and a depth of 423×. The DNBSEQ and Nanopore reads were quality-controlled by SOAPnuke V 1.5.6 (3) and porechop V 0.2.4, individually (4). A *k*-mer genome coverage of 39 × with a *k*-mer number of 15 was calculated by GCE (https://github.com/fanagislab/GCE). A circular chromosome of 4,761,538 bp was assembled with a coverage of 692 × and GC content of 61.5% by Canu V 1.5 (5). CDS prediction and annotation was performed by the NCBI Prokaryotic Genome Annotation Pipeline (PGAP) with GeneMarkS-2+ (6). A total of 4,340 genes including 4,286 CDS genes and 54 RNA-coding regions were predicted and annotated. Default parameters were used for all software.

## Data Availability

The whole-genome nucleotide sequence of *A. xenomutans* HF10 has been deposited in GenBank under the accession number CP136240. The SRA data of DNBSEQ library have been deposited under accession number SRX22500292. The Nanopore library SRA data have been deposited under accession number SRX22576803.
